# Combined effects of heavy metals and microplastics on maize grown in acid and alkaline soils inoculated with plant growth promoting rhizobacteria

**DOI:** 10.1371/journal.pone.0338112

**Published:** 2025-12-30

**Authors:** Asma Shabani, Reza Ghasemi-Fasaei, Mehdi Zarei, Sajjad Abbasi

**Affiliations:** 1 Department of Soil Science, School of Agriculture, Shiraz University, Shiraz, Iran; 2 Department of Earth Sciences, College of Science, Shiraz University, Shiraz, Iran; Manipal Academy of Higher Education (MAHE), INDIA

## Abstract

Microplastic particles (MPs) are recognized as novel pollutants, and their interactions with heavy metals (HMs) in soil can threaten ecosystem health and agricultural productivity. This study evaluates the combined effects of biodegradable polylactic acid (PLA) and non-biodegradable low-density polyethylene (LDPE) MPs and HMs include lead (Pb), cadmium (Cd), zinc (Zn), and nickel (Ni) on soil properties and maize growth. Maize was grown in alkaline (pH = 7.8) and acidic (pH = 5.6) soils under five MPs treatments (control, 1% LDPE, 5% LDPE, 1% PLA and 5% PLA) and Plant growth-promoting Rhizobacteria (PGPR) treatments including control, *Pseudomonas fluorescens* (*P. fluorescens*) and *Azospirillum lipoferum* (*A. lipoferum*) for 50 days. Results showed that PLA increased soil pH, while LDPE decreased it. Microplastic particles elevated electrical conductivity (EC) and dissolved organic carbon (DOC), with LDPE having a stronger effect on EC and PLA on DOC. LDPE enhanced HMs uptake in maize, whereas PLA decreased it. In alkaline soil, both MPs reduced plant biomass; however, reduction was not significant in PLA treatments. In contrast, in acidic soil, PLA increased shoot and root dry weights by 22.5% and 47.95%, respectively. The 5% LDPE treatment without bacteria caused the most significant reduction in shoot and root dry weights with decreases of 33.4% and 42.8% in alkaline soil; 26.8% and 21.1% in acidic soil respectively. PGPR increased soil DOC, improved plant growth, mitigated LDPE’s negative effects, and amplified PLA’s benefits. These findings highlight the importance of MP type, soil conditions, and PGPR for managing MPs-HMs contamination, with PLA and PGPR as sustainable agriculture strategies.

## 1. Introduction

Plastics are integral components of modern society because of their widespread use; however, a considerable share (approximately 99%) eventually enters the environment as waste [1]. Plastics gradually decompose into microplastics, which are particles smaller than 5 millimeters, and persist in soil due to their resistance to decomposition [2]. The accumulation of MPs in agricultural soils poses a substantial challenge, as it can threaten soil ecosystem health and functionality. Furthermore, exposure of crops to these residues may pose a potential risk to the safety and sustainability of the food chain [3]. MPs are classified into two groups: biodegradable and non-biodegradable [4]. Recently, concerns over the environmental impact of petroleum-based (non-biodegradable) plastics have prompted efforts to replace them with alternatives made from natural or biodegradable materials [5]. PLA is one of the leading materials in this shift, with increasing demand. As PLA production grows, its presence in plastic waste will also rise, highlighting the need to assess its ecological impact [6].

Soils are primarily contaminated with HMs in consequence of different anthropogenic activities, including industrial operations, agricultural practices, and transportation [7]. This contamination suggests that MPs and HMs can co-exist in the soil, maintaining its polluted state over long periods. The presence of MPs directly and indirectly influenced the environmental behavior and ecological threats of HMs. MPs can affect the adsorption and desorption processes of HMs, with adsorption capacity varying significantly based on the type and characteristics of both MPs and HMs [8–12]. For instance Chakraborty et al. [12] identified the type and concentration of MPs as two highly influential factors affecting the bioavailability of HMs in soil. Li et al. [11] showed that high concentrations of polyethylene (PE) (10%) reduced adsorption but increased desorption of Zn^2+^ and Pb^2+^ in soils, enhancing their bioavailability, while lower concentrations have no significant effect. Godoy et al. [8] observed that PE and polyvinyl chloride (PVC) show high adsorption capacities for Pb, Zn and chromium (Cr) whereas polyethylene terephthalate (PET) demonstrate relatively low adsorption capacities. Zou et al. [10] demonstrated that Pb has a stronger adsorption affinity to MPs than copper (Cu) and Cd, likely due to more intense electrostatic forces between Pb and MPs surface. The ability of MPs to adsorb is greatly affected by surface features, structure, porosity, particle size, and extent of degradation [8,9,13]. For example, smaller MPs with a larger specific surface area tend to show a higher adsorption potential [13]. Additionally, research indicates that the adsorption of elements such as arsenic (As), titanium (Ti), Ni, and Cd increases as MPs undergo aging and degradation [14,15]. Mao et al. [9] investigated the adsorption behavior of polystyrene (PS) toward several HMs before and after the aging process. Their findings revealed that the progressive aging of PS markedly enhanced its metal adsorption capacity, primarily due to the modifications in its surface chemistry and physicochemical characteristics. Yan et al. [16] found that biodegradable PLA exhibit a greater adsorption potential for HMs, including Cd, Cu, Pb, and Zn, compared to non-biodegradable PVC and PE.

MPs can change some of soil physicochemical properties included porosity, aggregates and water holding capacity [17]. Therefore, the bioavailability of HMs can be influenced by various biotic and abiotic soil factors, such as pH, DOC, soil aggregates, root secretions, and the microbial community. MPs have been shown to impact these factors, thereby indirectly affecting the environmental behavior of HMs [18–20]. For instance, Liu et al. [18] demonstrated that the addition of 2% PLA microplastics increased soil pH and modified micronutrient levels, thereby indirectly lowering Cd bioavailability in the soil–rice system. Nevertheless, studies on the effects of MPs on soil pH and DOC have produced inconsistent findings. Some studies have noted that MPs increase soil pH [20–22], while others report a decrease [11,23–25] or no significant impact [26]. Similarly, the influence of MPs on soil DOC varies, with some studies reporting an increase [25,27,28], others a decrease [24], and some finding no significant effect [29,30]. Li et al. [31] investigated the effect of MPs on Cd concentrations in plants across various soil types, observing contrasting results. In alkaline soil, both 1% and 0.1% MPs reduced Cd concentrations, while in acidic soil, only the higher dose (1%) caused a decrease in Cd concentrations. This variation was attributed to increased DOC and pH in acidic soil at the higher MPs dose. Wang et al. [25] reported that 10% PE in soil lowered pH and cation exchange capacity (CEC) while increasing DOC, resulting in increased Cd availability and concentration in plants. The role of MPs in HMs bioavailability remains debated. Several studies suggest that MPs can reduce HMs bioavailability and mitigate their negative effects on plants [32–34]. While, other researches indicate that MPs may enhance HMs toxicity and inhibit plant growth [11,25,35]. For instance, Zong et al. [33] showed that PS alleviated Cu and Cd toxicity in wheat seedlings, and Lian et al. [32] reported a similar reduction of Cd toxicity in wheat leaves by PS nano plastics.

Various strategies have been developed to enhance plant growth and mitigate damage and toxicity caused by environmental stressors. Among these, PGPR represent an eco-friendly and promising method for improving plant resilience under stressful conditions. PGPR can mitigate stress caused by HMs, promote plant growth, and influence HMs accumulation through a variety of processes [36]. *A. lipoferum* and *P*.*fluorescens* are well-characterized PGPR that enhance plant performance under abiotic stresses, including HMs contamination. Inoculation with *P. fluorescens* has been reported to improve root and shoot development, nutrient uptake, and phytohormone production, ultimately promoting overall plant growth under HMs stress [37]. Similarly, *A. lipoferum* contributes to root system development, nitrogen assimilation, and yield stability under adverse conditions [38]. These PGPR strains can also modulate HMs availability and mobility in the rhizosphere by altering soil chemistry, including pH, DOC, and redox potential, as well as by secreting bioactive compounds such as siderophores, extracellular polysaccharides, and organic acids [39]. Although these mechanisms may occasionally increase metal accumulation in plant tissues, the overall physiological tolerance is enhanced, resulting in improved growth and yield [37,38].

However, their role in addressing combined pollutants remains underexplored. Recent studies by [40,41] confirmed PGPR’s effectiveness in reducing the negative effects of PE and Cd contamination on sorghum growth. Similarly, Zhang et al. [42] showed that PGPR strains isolated from soil significantly alleviated stress caused by combined Cd and PVC contamination, enhancing sorghum biomass.

Iran, the 18th largest and most populous country globally, consumes approximately half a million tons of plastic annually, ranking fifth in the world for high plastic consumption [43]. Around 5,000 tons of plastic waste are deposited in landfills daily [44]. Despite this extensive consumption, research on the effects of MPs on soil properties and agricultural plants in Iran is scarce. Moreover, global studies have predominantly concentrated on the impact of MPs in a single soil type, while, soil type can significantly influence the varying and, at times, contradictory effects of MPs. Additionally, little research examines the combined impact of multiple HMs under MPs contamination on soil properties. Based on the above, this project aims to achieve the following objectives:

- To examine the effects of two types of MPs with distinct characteristics (biodegradable and non-biodegradable) on selected soil properties.
- To investigate the impact of the simultaneous presence of multiple HMs under MPs contamination on soil characteristics and maize growth.
- To assess the impacts of two PGPR species, *P. fluorescens* and *A. lipoferum*, under combined HMs and MPs contamination conditions.

## 2. Materials and method

### 2.1. Soil, plant, and MPs

In this study, two soil types were utilized: an alkaline soil (pH = 7.8) from the Agricultural Research Station of Shiraz University, Fars Province, Southern Iran, and an acidic soil (pH = 5.6) from tea farms in Lahijan County, Gilan Province, Northern Iran. Both soil samples were collected from a depth of 0–30 cm. After collection, soils were air-dried, organic residues removed, and sieved to 2 mm for analysis and pot cultivation. Briefly, pH and EC were measured in saturated paste extract. Soil organic matter and texture were measured using the acid digestion method [45] and the Bouyoucos hydrometer method [46], respectively. The calcium carbonate equivalent (CCE) was assessed by calcimeter method [47] and the CEC was determined using Bower’s method (the ammonium acetate method pH = 7.0) [48]. Available form of Cd, Pb, Zn, and Ni in soil samples were extracted by DTPA extraction method [49], and their concentration was determined by atomic absorption spectroscopy (Shimadzu AA 670G, Japan). The physical and chemical characteristics of both soil types are provided in Table 1.

**Table 1:** Some physical and chemical properties of soil.

	Sand	Silt	Clay	Texture	pH	EC	%OM	CEC	CCE	Pb	Cd	Zn	Ni
Soil	---------%---------			–	–	ds m^-1^	%	Cmol_(+)_ kg^-1^	%	----------mg. kg^-1^ ----------			
Alkaline	31.4	27.2	41.4	Clay loam	7.8	0.85	0.68	14	41.2	0.28	0.18	0.68	0.15
Acidic	51.3	25.3	23.4	Sandy clay loam	5.6	0.47	2.58	20	1.75	1.93	0.24	2.76	0.61

EC and pH measured in saturated paste extract.

OM: Organic matter.

CEC: cation exchange capacity.

CCE: Calcium Carbonate Equivalent.

Exchangeable concentration of Pb, Cd, Zn and Ni extracted by DTPA.

Two common types of MPs were selected: LDPE, a petroleum-based, non-biodegradable polymer, and PLA, a biodegradable polyester derived from renewable resources such as starch [50]. The densities of LDPE and PLA were measured as 0.92 g cm^-3^ and 1.24 g cm^-3^, respectively. Key characteristics of these polymers, including their functional groups and morphologies, were analyzed using Fourier Transform Infrared Spectroscopy (FTIR; AVATAR, Thermo, USA) and Scanning Electron Microscopy (SEM; MIRA III, TESCAN, Czech Republic), respectively. Nitrogen adsorption–desorption measurements were carried out at 77 K to characterize the surface properties and pore structure of the PLA and LDPE samples. The obtained isotherm data were analyzed using the Brunauer–Emmett–Teller (BET) model to determine the specific surface area and adsorption energy constant (C). Furthermore, the Barrett–Joyner–Halenda (BJH) model was employed to calculate the total pore volume and mean pore diameter from the adsorption branch of the isotherm [51].

MPs between 0.5–1 mm were selected after sieving because this size is commonly found in agricultural soils [52–54], and allows for uniform soil mixing and effective interaction with soil particles and plant roots. Larger particles (>1 mm) act more like microplastics with limited root interaction, while smaller ones could enter plant tissues, which was outside the study’s objectives. Prior to use, the MPs were thoroughly washed with 0.1 mol L ⁻ ¹ HCl, and rinsed with distilled water. Maize seeds (Single Cross 715 variety) were procured from the Seed and Plant Improvement Institute of Iran. The bacterial inoculum liquid (10⁷ CFU/mL) consisted of *A. lipoferum* (collection code CCSM-B00232) and *P. fluorescens* (collection code CCSM-B00162), both obtained from the Soil and Water Research Institute of Iran. Both strains have been widely applied in Iranian cropping systems, especially corn and wheat. Their nativeness, environmental adaptability, and prior experimental evidence led to their selection for this study to evaluate their role in mitigating combined stress from HMs and MPs.

To ensure seed disinfection, the seeds were immersed in a 2% sodium hypochlorite solution for 15 minutes, then rinsed multiple times with distilled water.

### 2.2. Treatments and experimental design

Before initiating the experiment, the soil in each pot sieved through a 2 mm mesh, was separated into four equivalent parts. Each part was polluted with 100 mg/kg of four HMs (Pb, Cd, Ni, and Zn) using their nitrate salts. After thorough mixing to ensure uniform metal distribution, the samples were incubated for one month at 25 ± 2°C while maintaining soil moisture at field capacity [55] (The final concentration of each metal was 100 mg/kg, i.e., a total metal concentration of 4 × 100 mg/kg was present in each pot). Subsequently, different types of MPs were added to the HMs-contaminated soils according to the experimental treatments and incubated for three weeks. The experiment followed a factorial study in a completely randomized design with three replications, conducted separately for acidic and alkaline soils. Treatments included five concentrations of MPs: CK: control without MPs, 1% and 5% LDPE, 1% and 5% PLA and three types of microbial inoculation: CK: control without inoculation, inoculation with *P. fluorescens*, and inoculation with *A. lipoferum*. A total of 45 experimental units (5 microplastic levels × 3 microbial treatments × 3 replicates) were established for each soil type, and 90 pots were evaluated overall across both acidic and alkaline soils. Each pot contained 1 kg of soil, and essential nutrients were added before planting based on soil test results. Ten sterilized seeds were sown per pot, and one week after germination, weaker seedlings were removed, leaving three seedlings per pot. Bacterial inoculation was performed at planting by applying 2 mL of inoculum per seed. During the entire growth period soil moisture was sustained at field capacity.

### 2.3. Soil and plant analysis

One day before harvest, a SPAD-502 chlorophyll meter was used to measure leaf greenness. Readings were taken from the middle leaf position, and the final SPAD value was calculated as the average of five measurements. The device provides dimensionless readings in SPAD units, which represent a relative index for non-destructive estimation of leaf greenness. The instrument was calibrated before each measurement through the standard calibration procedure recommended by the manufacturer, following the manufacturer’s instructions.

Leaf relative water content (RWC) and membrane stability index (MSI) were also assessed in fresh leaves. For each treatment, RWC was determined using the method of González and González-Vilar, [56]. Briefly, five-leaf discs (1 cm in diameter) were taken from the most recently developed leaf. After measuring their fresh weight (FW), the discs were placed in distilled water in a refrigerator for 4 hours until fully swollen. The turgid weight (TW) was then recorded. Subsequently, the samples were dried in an oven at 70°C for 48 hours, and their dry weight (DW) was measured. RWC was calculated using the following equation.


$$\text{RWC}\;(\%)=\left(\frac{FW-DW}{TW-DW}\right)*\text{100}$$

Cell membrane stability is a potential indicator of MPs stress response [57]. To assess membrane damage, MSI was measured. Following the method of Sairam et al. [58], two sample groups were prepared. In each group, 0.1 g of fresh, healthy aerial plant tissue was placed in 10 mL of double-distilled water. The first group’s test tubes were incubated in a water bath at 40°C for 30 minutes (C_1_), while the second group’s tubes were heated to 100°C for 15 minutes (C_2_). After cooling, the electrical conductivity of each sample was measured. MSI was then calculated using the following equation.


$$\text{MSI}\;(\%)=[\text{1}-\left(\frac{C_{\text{1}}}{C_{\text{2}}}\right)*\text{100}$$

After the 50-day cultivation, plants were harvested, separated into shoots and roots, washed, and oven-dried at 65°C for 72 hours. Samples underwent wet digestion with HCl, HNO3 (1:3), and 30% H_2_O_2_ on an electric heating plate [59]. The concentrations of HMs were quantified using an atomic absorption spectrophotometer (Shimadzu AA 670G, Japan). The transport factor (TF) for HMs, signifying the movement from roots to aerial shoots, was calculated accordingly. Furthermore, the pH and EC of the potting soil were measured using a soil-to-water ratio of 1:2.5, while DOC was assessed at a 1:5 soil-to-water ratio using a spectrophotometer set at a wavelength of 254 nm [60].

All experimental procedures and analytical measurements were conducted under stringent quality assurance and quality control (QA/QC) protocols to ensure the accuracy, reliability, and reproducibility of the results. Soil and plant samples were collected, homogenized, and analyzed in triplicate to minimize random errors. Plants were cultivated under controlled greenhouse conditions, and all treatments were applied uniformly across replicates. Prior to the experiments, the microplastic polymers were characterized by FTIR spectroscopy to verify their chemical composition and purity. Measurements of pH, EC and DOC were performed using properly calibrated instruments with standard and blank buffer solutions. For the determination of HMs concentrations, an atomic absorption spectrophotometer was calibrated using certified standard solutions before each analytical run. Instrumental drift and baseline stability were checked every ten samples using a mid-level standard, while blank and reference samples were routinely analyzed to confirm data accuracy and rule out contamination. Method detection limits, analytical precision, and recovery rates were all within acceptable quality control ranges (RSD < 5%). To prevent trace metal contamination, all glassware and plastic ware were thoroughly acid-washed with 10% nitric acid and rinsed twice with deionized water prior to use.

### 2.4. Statistical analysis

Data normality and variance homogeneity were tested using the Shapiro–Wilk and Levene’s tests, respectively. Two-way ANOVA was then performed to assess the main and interactive effects of microplastic treatment and microbial inoculation. Mean differences were evaluated using Duncan’s multiple range test at p ≤ 0.05. Exact p-values, F-values, and effect sizes for alkaline and acidic soils are reported in S1 and S2 Tables. All statistical analyses were carried out using SAS 9.4, and graphical representations were prepared using Origin 2021.

## 3. Results

### 3.1. Characteristics of MPs

**Fig 1:**
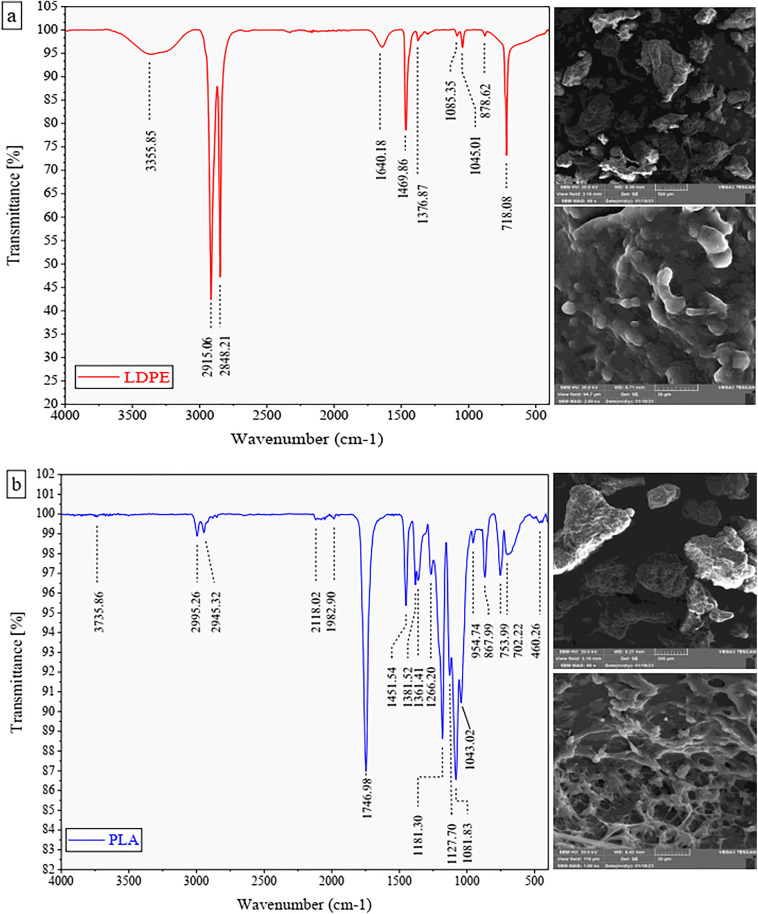
Fourier Transform Infrared Spectroscopy (FTIR) spectra and Scanning Electron Microscopy (SEM) images of the surface microplastic particles of the a) low-density polyethylene (LDPE) and b) polylactic acid (PLA).

SEM images revealed distinct differences in the surface morphology of LDPE (Fig 1A) and PLA (Fig 1B). The surface of PLA exhibited greater roughness, characterized by pronounced folds and indentations, whereas, LDPE displayed a comparatively smoother surface.

The functional groups of MPs were characterized using FTIR spectroscopy (Fig 1). For LDPE (Fig 1A), four characteristic peaks were observed at wavelengths of 2915.06, 2848.21, 1469.86, and 718.08 cm^-1^, corresponding to CH_2_ stretching, CH_2_ stretching, CH_2_ bending, and CH_2_ rocking vibrations, respectively [61]. Similarly, four main peaks were identified for PLA (Fig 1B) at wavelengths of 1746.98, 1181.30, 1081.83, and 1043.02 cm^-1^. These peaks represent the functional groups C = O, -C-O- in -CH-O-C, -C-O- in -O-C = O, and -C-O- in -O-C = O, respectively [62].

Based on the results presented in S3 Table, PLA exhibited a higher specific surface area (1.32 vs. 1.01 m^2^·g^-1^ for LDPE), a larger total pore volume (0.0836 vs. 0.0427 cm^3^·g^-1^), and a smaller mean pore diameter (103.16 vs. 186.61 nm). In addition, the BET constant was notably higher for PLA (C = 10.31 vs. 6.29), suggesting stronger adsorption energy between the adsorbate and the polymer surface. These characteristics indicate that PLA possesses a more developed mesoporous structure, a higher density of active sites, and greater surface adsorption potential. The BJH analysis further confirmed the enhanced accessible surface area of PLA (6.66 vs. 3.52 m^2^·g^-1^), implying that PLA microplastics are more capable of interacting with environmental molecules such as HMs. In contrast, LDPE, with its lower surface area and pore volume, exhibited a weaker adsorption capacity [63,64].

### 3.2. Chemical properties of soil

**Fig 2:**
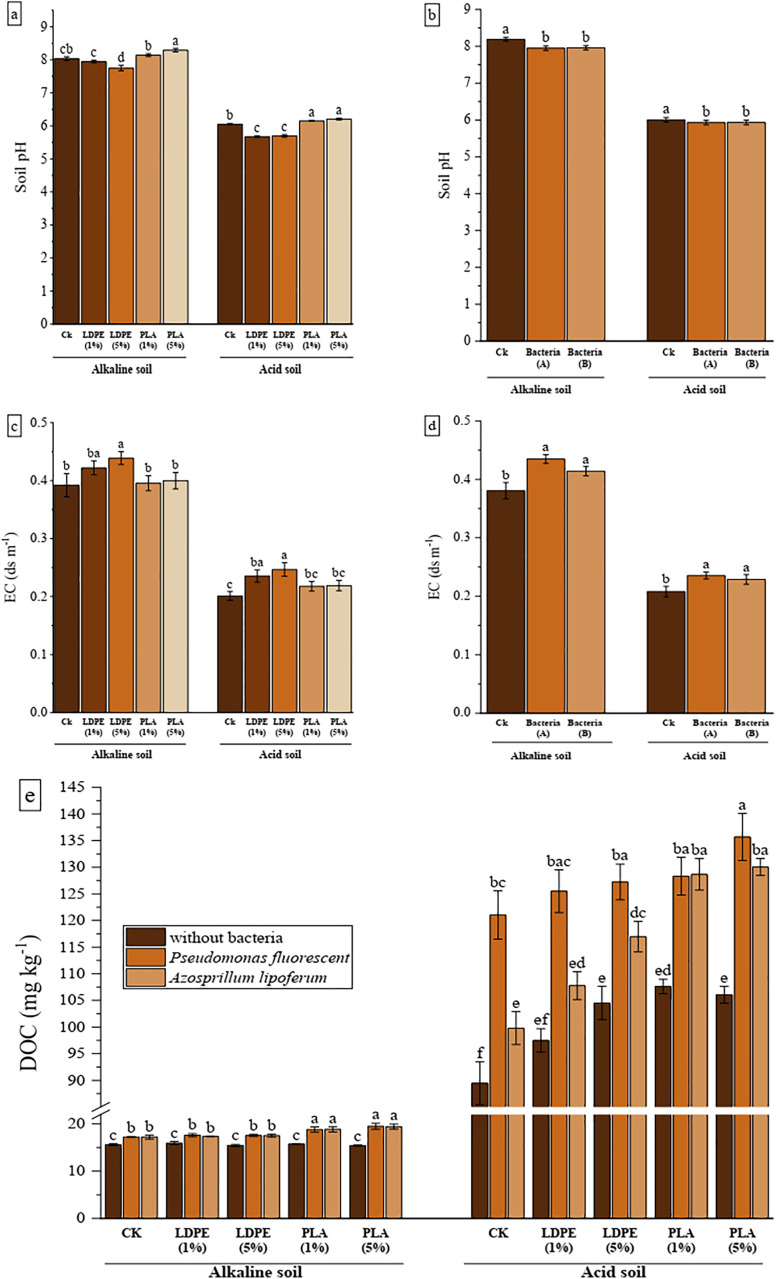
The effect of MPs type and concentration (A) and bacteria (B) on soil pH, EC and DOC in acidic and alkaline soils. CK: without MPs, LDPE: low-density polyethylene, PLA: polylactic acid, bacteria A: *P. fluorescens*, bacteria B: *A. lipoferum.* The distinct letters on the bars denote statistically significant differences between the means, as determined by a two-way ANOVA followed by Duncan’s multiple range test (P < 0.05). (Values are presented as means ± SE, n = 3).

Chemical properties of the soil, including pH, EC, and DOC mainly influenced by varying levels of MPs and PGPR (Table 2, Fig 2). As shown in Fig 2A, PLA and LDPE had contrasting effects on soil pH in both alkaline and acidic soils. PLA increased soil pH, whereas LDPE decreased it. In alkaline soil, the highest pH was detected in the 5% PLA treatment, showing a 3.21% increase compared to the control (CK), whereas the lowest pH was observed under the 5% LDPE treatment, with a 3.54% decrease (P < 0.001). In acidic soil, PLA treatments led to an average pH increase of 2.12%, whereas LDPE treatments resulted in a 6.01% reduction relative to the CK (P < 0.001). In both soil types, the presence of *P. fluorescens* and *A. lipoferum* bacteria led to a reduction in pH compared to the CK (Fig 2B). This reduction averaged 2.88% in alkaline soil (P < 0.001) and 1.16% in acidic soil (P < 0.05).

**Table 2:** Effects of MPs type and concentration (A), bacteria (B) and their interactions on measured parameters based on a two-way ANOVA analysis.

		----------------------------- Alkaline soil -----------------------------								---------------------------- Acidic soil ---------------------------							
Source		A		B		A × B		Error	C.V	A		B		A × B		Error	C.V
df		4		2		8		30	–	4		2		8		30	–
		**Mean Square**															
Shoot dry weight		4.58	***	9.62	***	0.38	*	0.17	8.54	7.52	***	6.59	***	0.32	*	0.13	5.82
Root dry weight		0.77	***	1.78	***	0.05	*	0.02	8.79	1.21	***	1.25	***	0.12	*	0.05	9.79
Chl		10.94	*	517.19	***	2.38	ns	2.93	4.90	58.14	***	41.49	*	0.57	ns	8.10	7.72
MSI		702.23	***	133.40	**	16.60	ns	20.24	6.08	520.66	***	9.18	ns	2.38	ns	20.68	5.56
RWC		459.97	***	109.41	*	10.86	ns	29.44	7.41	332.88	***	19.00	ns	6.50	ns	27.02	6.45
SD		0.91	**	1.40	**	0.10	ns	0.18	5.36	3.45	***	0.65	ns	0.04	ns	0.24	5.31
Soil	DOC	3.65	***	30.45	***	1.16	*	0.41	3.69	629.85	***	2667.82	***	70.13	*	30.16	4.77
	EC	0.004	*	0.01	***	0.002	ns	0.001	8.07	0.003	*	0.003	*	0.0006	ns	0.001	12.14
	pH	0.37	***	0.28	***	0.004	ns	0.02	1.72	0.58	***	0.03	*	0.0007	ns	0.006	1.28
TF	Pb	0.01	*	0.02	**	0.004	ns	0.003	12.03	0.03	*	0.014	ns	0.006	ns	0.009	14.49
	Cd	0.03	***	0.02	**	0.004	ns	0.003	12.42	0.003	ns	0.0001	ns	0.001	ns	0.001	14.15
	Zn	0.004	ns	0.04	**	0.001	ns	0.006	12.30	0.04	**	0.007	ns	0.004	ns	0.009	12.34
	Ni	0.02	ns	0.04	**	0.002	ns	0.006	13.08	0.002	ns	0.008	*	0.002	ns	0.002	11.77
Pb	Shoot	37.15	***	13.59	***	1.07	ns	0.95	8.88	90.30	***	23.31	**	7.70	*	2.80	9.02
	Root	104.17	***	2.22	ns	0.78	ns	3.38	7.83	331.77	***	0.82	ns	0.92	ns	8.49	10.03
Cd	Shoot	410.34	***	16.38	ns	7.54	ns	7.04	7.67	2247.81	***	298.29	**	19.47	ns	43.97	7.96
	Root	495.46	***	843.49	***	43.96	ns	41.09	8.20	37294.55	***	4822.12	*	1417.37	ns	970.95	9.11
Zn	Shoot	176.55	**	317.96	***	12.38	ns	32.86	10.05	682.09	***	59.05	ns	10.13	ns	23.55	8.26
	Root	690.26	***	3.35	ns	7.78	ns	47.64	7.87	1327.30	***	349.12	**	37.95	ns	51.71	9.10
Ni	Shoot	40.13	***	0.61	ns	0.54	ns	1.51	7.11	355.65	***	59.96	ns	11.67	ns	24.85	8.99
	Root	239.88	***	94.47	***	5.173	ns	6.56	8.83	4488.53	***	3298.95	***	273.03	ns	258.24	9.94

Abbreviations: A: MPs (type and concentration), B: bacteria, MSI: membrane stability index, RWC: relative water content, DOC: dissolved organic carbon, EC: electrical conductivity; TF; transfer factor; Chl: chlorophyll (SPAD reading); SD: stem diameter; ns: non-significant,* p < 0.05, ** p < 0.01, and *** p < 0.001.

Across all treatments and soil types, the trend in EC showed an increase relative to the CK (Fig 2C). The 5% LDPE treatment resulted in the highest EC values, with increases of 11.90% and 22.65% in alkaline and acidic soils, respectively (P < 0.05). Similarly, bacterial treatments contributed to an increase in soil EC, with average increases of 11.47% in alkaline soil (P < 0.001) and 11.54% in acidic soil (P < 0.05) (Fig 2D).

The interactions between MPs and PGPR were statistically significant for the DOC content in the soil (P < 0.05). As shown in Fig 2E, in both soil types, the application of PGPR enhanced DOC levels under both conditions: HMs-contaminated (CK) and co-contaminated with both HMs and MPs. In alkaline soil, DOC content did not vary significantly without PGPR. However, in the presence of PGPR, the DOC content in PLA treatments increased significantly compared to CK, with average increases of 11.24% and 11.13% observed for *P. fluorescens* and *A. lipoferum*, respectively. In acidic soil, the DOC content increased across various treatments (except for the 1% LDPE) in the absence of PGPR. The greatest rise in DOC content was noted in the 5% PLA treatment, with increase of 12.08% and 30.03% in the presence of *P. fluorescens* and *A. lipoferum*, respectively, relative to CK.

### 3.3. Plant growth indicators

**Fig 3:**
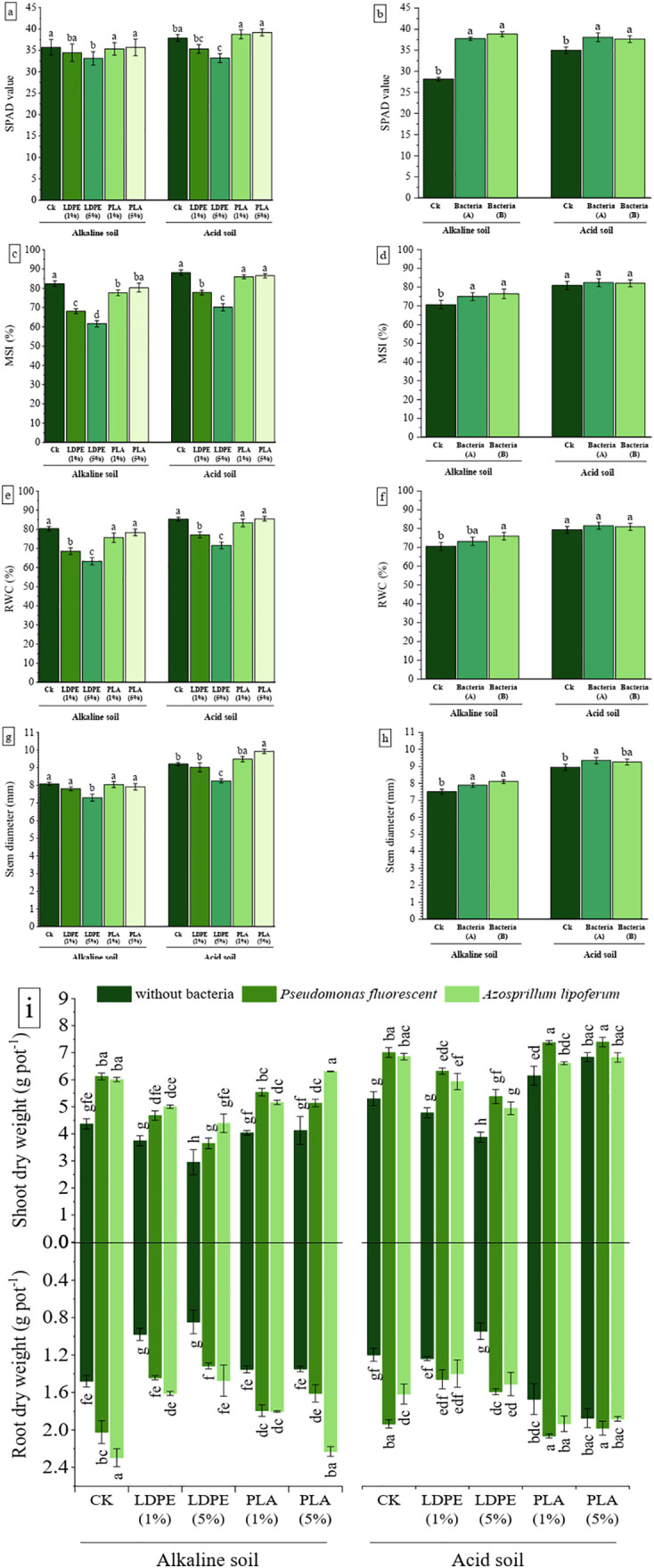
The effect of MPs type and concentration (A) and bacteria (B) on the dry weight of shoots and roots, SPAD, MSI (membrane stability index), RWC (relative water content) and stem diameter of maize grown in acidic and alkaline soil. CK: without MPs, LDPE: low-density polyethylene, PLA: polylactic acid, bacteria A: *P. fluorescens*, bacteria B: *A. lipoferum.* The distinct letters on the bars denote statistically significant differences between the means, as determined by a two-way ANOVA followed by Duncan’s multiple range test (P < 0.05). (Values are presented as means ± SE, n = 3).

Fig 3A shows that the lowest chlorophyll index occurred in the 5% LDPE treatment, with reductions of 7.26% in alkaline soil (P < 0.05) and 12.43% in acidic soil (P < 0.001) compared to the CK. In alkaline soil, all treatments indicated a declining pattern in the chlorophyll index, with the most significant reductions in LDPE treatments. Conversely, in acidic soil, PLA treatments slightly increased the chlorophyll index compared to CK, though the increase was not statistically notable. The use of PGPR notably improved the chlorophyll index in both alkaline (P < 0.001) and acidic (P < 0.05) soils (Table 2, Fig 3B). In alkaline soil, *P*. *fluorescens* and *A*. *lipoferum* increased the chlorophyll index by 34.02% and 37.99%, respectively, while in acidic soil, the increases were 8.79% and 7.55%.

RWC and MSI are key indicators for assessing plant stress induced by MPs. MPs promote reactive oxygen species (ROS) production, leading to oxidative damage to membrane lipids [65]. The various levels of MPs significantly influenced MSI and RWC in both soils (P < 0.001) (Table 2). In alkaline soil, both types of MPs reduced the MSI compared to the CK (Fig 3C), while the RWC was only decreased by LDPE (Fig 3E). In acidic soil, PLA had no effect on either MSI or RWC, whereas LDPE caused a reduction in both indices (Fig 3C and 3E). The impact of *P. fluorescens* and *A. lipoferum* on the MSI (P < 0.01) and RWC (P < 0.05) was significant in alkaline soil but not in acidic soil (Table 2). In alkaline soil, *P. fluorescens* and *A. lipoferum* increased MSI by 6.10% and 8.10%, and RWC by 3.71% and 7.66%, respectively, compared to the CK (Fig 3D and 3F).

As demonstrated by Fig 3G, in alkaline soil, a considerable decline in stem diameter was observed in the 5% LDPE treatment, with a decrease of 9.75% compared to the CK (P < 0.01). In acidic soil, the 5% PLA treatment resulted in the highest stem diameter, showing an increase of 7.69%, while the 5% LDPE treatment led to the lowest stem diameter, with a decrease of 10.43% relative to CK (P < 0.001). In alkaline soil, the use of PGPR resulted in a notable increase in stem diameter compared to the CK (P < 0.01) (Fig 3H). Treatments with *P. fluorescens* and *A. lipoferum* resulted in increases of 32.5% and 8.2%, respectively. Conversely, in acidic soil, the analysis of variance (Table 2) showed that the main effect of PGPR on stem diameter was not significant.

According to ANOVA results (Table 2), interactions between different levels of MPs and PGPR significantly affected shoot and root dry weights in both soils (P < 0.05). In alkaline soil, both LDPE and PLA reduced plant biomass, though the decrease was not significant in PLA treatments. The greatest reductions in shoot and root dry weights occurred in the 5% LDPE treatment without bacterial inoculation, with decreases of 33.4% and 42.8%, respectively, compared to CK (Fig 3I). In acidic soil, the absence of bacteria in the 5% LDPE treatment led to the most significant biomass reductions, with shoot and root dry weights decreasing by 26.8% and 21.1%, respectively, relative to CK. In contrast, PLA treatments increased shoot and root dry weights by an average of 22.5% and 47.95%, respectively. In alkaline soil, *P. fluorescens* increased shoot dry weight by 24.6–40.2% and root dry weight by 19.26–55.51%, while *A. lipoferum* led to increases of 27.8–48.6% in shoot dry weight and 33.25–74% in root dry weight, compared to CK. In acidic soil, *P. fluorescens* enhanced shoot dry weight by 8.2–38.7% and root dry weight by 7.5–61.6%, while *A. lipoferum* increased shoot dry weight by 7.2–29.4% and root dry weight by 0.4–7.59%. Unlike LDPE, the combined contamination of HMs and PLA did not negatively impact PGPR performance in acidic soil. This effect was especially notable in root dry weight, which increased by 4.45% with *P. fluorescens* and 18% with *A. lipoferum* under PLA and HMs contamination, compared to CK.

### 3.4. Cd, Zn, Pb, and Ni concentrations in plant tissue

**Fig 4:**
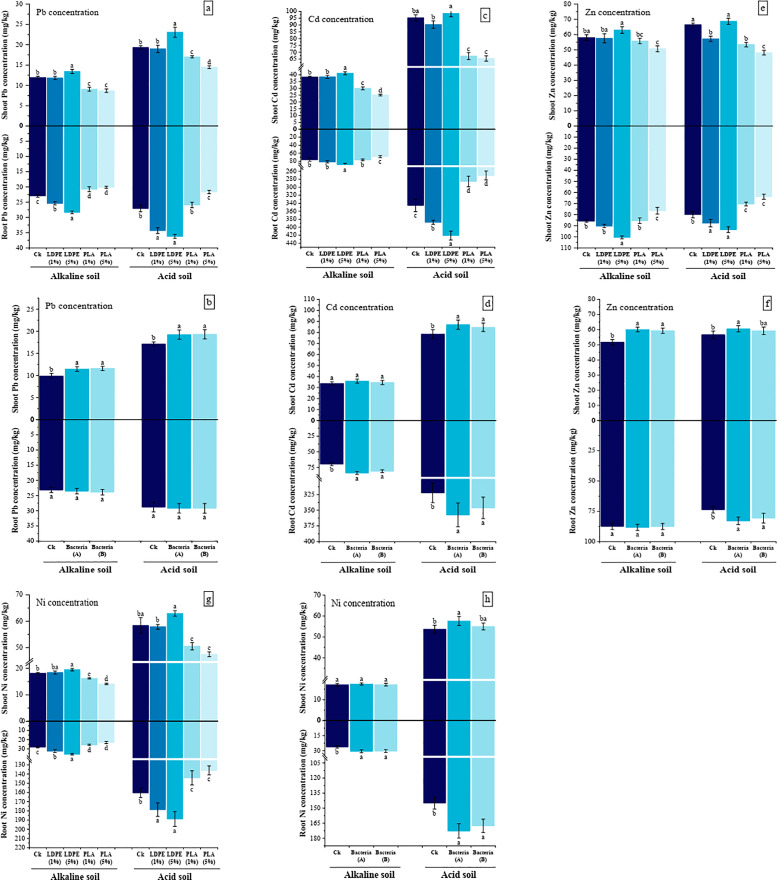
The effect of MPs type and concentration (A) and bacteria (B) on Pb, Zn, Cd and Ni concentrations in shoot and root of maize grown in acidic and alkaline soil. CK: without MPs, LDPE: low-density polyethylene, PLA: polylactic acid, bacteria A: *P. fluorescens*, bacteria B: *A. lipoferum.* The distinct letters on the bars denote statistically significant differences between the means, as determined by a two-way ANOVA followed by Duncan’s multiple range test (P < 0.05). (Values are presented as means ± SE, n = 3).

In both soil types, MPs significantly affected metal concentrations in the shoot (P < 0.001, except for Zn in alkaline soil) and root (P < 0.001). The results showed that in both soils, application of 5% LDPE led to the highest Pb (Fig 4A), Cd (Fig 4C), Zn (Fig 4E) and Ni (Fig 4G) concentrations in both shoot and root. In alkaline soil, shoot metal concentrations increased by 37.12% (Pb), 41.7% (Cd), 53.8% (Zn), and 48.7% (Ni), while root concentrations increased by 59.23% (Pb), 30.16% (Cd), 20.17% (Zn), and 48.27% (Ni). In acidic soil, shoot metal concentrations increased by 46.19% (Pb), 41.3% (Cd), 39.3% (Zn), and 81.7% (Ni), while root concentrations increased by 72.33% (Pb), 88.21% (Cd), 97.16% (Zn), and 66.17% (Ni). Polylactic acid (5%) resulted in the lowest metal concentrations in both shoot and root. In alkaline soil, shoot metal concentrations decreased by 33.27% (Pb), 45.34% (Cd), 65.12% (Zn), and 22.05% (Ni), while root concentrations decreased by 12.30% (Pb), 10.11% (Cd), 10.89% (Zn), and 17.93% (Ni). In acidic soil, shoot metal concentrations decreased by 25.06% (Pb), 31.34% (Cd), 27.65% (Zn), and 18.64% (Ni), while root concentrations decreased by 20.03% (Pb), 21.59% (Cd), 20.19% (Zn), and 15.25% (Ni).

The application of PGPR in alkaline soil led to average increases of 63.16% and 37.15% (P < 0.001) in the concentrations of Pb (Fig 4B) and Zn (Fig 4F) in the shoots, respectively, nevertheless, no notable changes were detected in the concentrations of Pb and Zn in the roots. Additionally, although the concentrations of Cd (Fig 4D) and Ni (Fig 4H) in the shoots were not affected by PGPR, their concentrations in the roots increased by an average of 18.3% and 16.84% (P < 0.001), respectively, compared to the CK. In acidic soil PGPR increased the concentrations of Cd, Zn, and Ni in the roots by averages of 16.9% (P < 0.05), 48.17% (P < 0.01), and 77.10% (P < 0.001), respectively, but no significant increase was observed in the concentration of Pb in the roots. Similarly, the concentrations of Cd and Pb in the shoots increased by averages of 9.4% and 12.6% (P < 0.01), respectively, in the presence of these bacteria. While *A. lipoferum* had no considerable impact on the concentrations of Zn and Ni in the shoots, *P. fluorescens* increased shoot Zn and Ni concentrations by 9.6% and 32.7%, respectively.

### 3.5. Metals transfer factor (TF)

**Fig 5:**
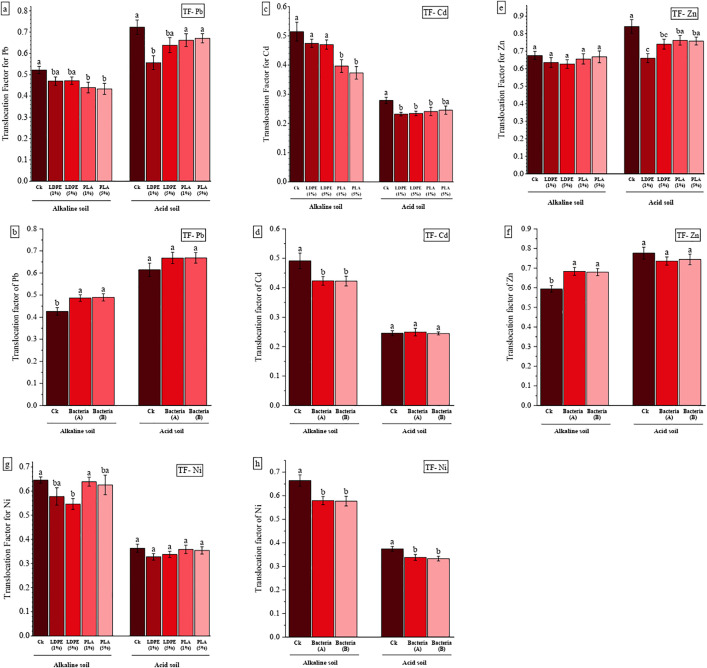
The effect of MPs type and concentration (A) and bacteria (B) on transport factor (TF) of Pb, Zn, Cd and Ni for maize grown in acidic and alkaline soil. CK: without MPs, LDPE: low-density polyethylene, PLA: polylactic acid, bacteria A: *P. fluorescens*, bacteria B: *A. lipoferum.* Different letters on the bars indicate significant differences among means using a two-way ANOVA followed by Duncan’s multiple range test (P < 0.05). (Means ± SE, n = 3).

The transport factor (TF) reflects the plant’s ability to transfer HMs from roots to shoots and leaves. The presence of MPs in both soil types reduced the TF across all treatments (Fig 5). However, these reductions were statistically significant only for the TF values of Pb (P < 0.05) and Cd (P < 0.001) in alkaline soil and those of Pb (P < 0.05) and Zn (P < 0.01) in acidic soil (Table 2). The lowest TF values for Pb (Fig 5A) and Cd (Fig 5C) in alkaline soil were associated with PLA treatments, which exhibited average reductions of 16.38% and 25.16%, respectively, compared to the CK. In acidic soil, the lowest TF values for Pb and Zn (Fig 5E) were observed in LDPE treatments, resulting in reductions of 17.35% and 16.77%, respectively, relative to the CK.

In alkaline soil, the effect of PGPR on the TF was significant (P < 0.01) (table 2), but the pattern of change varied among metals. The use of PGPR increased the TF of Pb (Fig 5B) and Zn (Fig 5F) by 14.63% and 14.7%, while decreasing the TF of Cd (Fig 5D) and Ni (Fig 5H) by 13.9% and 13.04%, respectively, compared to the Ck. In acidic soil, PGPR had no significant effect on TF, except for Ni (P < 0.05), where the TF decreased by 10.5% relative to the CK.

### 3.6. Correlation analysis in the soil-plant system

**Fig 6:**
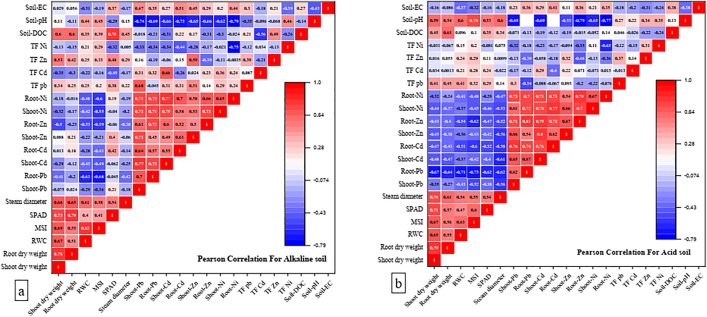
The correlation matrix heatmap for alkaline (A) and acidic soil (B) that shows the values of the Pearson correlation coefficient for all studied parameters. Blue color indicates the negative correlation and red color indicates the positive correlation.

Fig 6 illustrates the correlation between HMs concentrations in the shoots and roots of the plant, plant growth characteristics, and soil properties. Among various soil characteristics, pH exhibited the strongest correlation with metal concentrations in plants. Specifically, a decrease in soil pH was associated with an increase in metal accumulation in plant tissues. In both soil types, after pH, soil EC was identified as the second most influential factor, showing a positive correlation with metal concentrations in plant tissues. Among plant growth parameters, shoot and root dry weight, MSI, RWC, chlorophyll index, and stem diameter were negatively correlated with metal concentrations in both soils. Notably, these negative correlations were more evident in the acidic soil than in the alkaline soil.

## 4. Discussion

### 4.1. The effect of co-contamination of MPs and HMs on soil properties

In alkaline soil, MPs significantly altered soil pH only at higher concentrations (5%), while lower concentrations (1%) had minimal effect. In acidic soil, there was no significant difference among the concentrations of MPs (1% and 5%) in terms of their effect on soil pH. In this soil type, the effect of MPs on pH was mainly determined by the type of polymer, rather than its concentration. In both soil types, pH decreased with the addition of LDPE and increased with PLA. Previous studies have also shown a decrease in soil pH with PE contamination [25,66–68].

For example, increasing PE in Cd-contaminated soils lowered pH by 0.7% to 1.2% [25] and application of 2.5% and 5% PE reduced pH by 2.9% and 5.4%, respectively [69]. Rong et al. [70] reported that LDPE can alter the quantity of nitrifiers, which in turn can lower soil pH by releasing H^+^ ions. In contrast, PLA increased pH by decreasing nitrifier abundance [71]. The noted rise in pH when exposed to PLA was confirmed in our study and aligned with the results of Feng et al. [27], Liu et al. [18], Wang et al. [72] and Xu et al. [73]. When exposed to MPs, EC increased in both soil types, with more pronounced changes in acidic soil. The results also indicated a greater impact of LDPE compared to PLA on soil EC. The average increases in EC compared to control, in the presence of LDPE and PLA, were 9.77% and 1.41%, in alkaline soil and 19.89%% and 8.56% in acidic soil respectively.

Palansooriya et al. [66] attributed the increase in soil EC to the enhanced microbial activity in the presence of LDPE, which accelerated the degradation of organic matter and released various ions. Zhao et al. [30] reported an increase in EC in the presence of combined PE-Cd and PS-Cd contamination in a red soil (pH = 5.21). Li et al. [31] stated that the increase in soil EC resulting from the presence of MPs might be attributed to an increase in soil carbon source, which enhances the mobility of multiple ions.

In both soil types, PGPR decreased soil pH and increased EC, with no significant differences between bacterial types. Li et al. [74] and Abdollahi et al. [75] reported similar findings, attributing the reduction in soil pH to the secretion of organic acids by PGPR. They also noted that PGPR enhance microbial and enzymatic activities, facilitate the dissolution of insoluble phosphorus and potassium compounds, and contribute to nitrogen fixation. These factors collectively result in a rise in soil nutrient levels and, consequently, a rise in EC.

In the absence of bacteria, MPs had no significant impact on soil DOC in alkaline soil, but in acidic soil, all MPs treatments increased DOC, compared to control. This indicates that the influence of MPs on enhancing DOC was more evident in acidic conditions. The application of PGPR increased soil DOC in both soil types, regardless of MP presence. In alkaline soil, both bacterial types performed similarly, while in acidic soil, *P. fluorescens* was more effective than *A. lipoferum*. Furthermore, in both soils, PLA treatments led to a greater increase in DOC levels in the presence of bacteria. Several studies have emphasized the impact of biodegradable MPs, like PLA, on enhancing soil DOC. Research by Li et al. [31] and Soo et al. [76] showed that PLA carbon compared to PE is more easily broken down by microorganisms into water-soluble polymers, which are then used as a carbon source by microorganisms, resulting in a rise in soil DOC. Similarly, Sun et al. [77] also observed stronger effects on DOC from biodegradable MPs, such as poly butylene succinate (PBS) and PLA, compared to non-biodegradable MPs, like PE and PS.

Chen et al. [78], Feng et al. [27], and Wang et al. [79] reported an increase in DOC concentration following the addition of 2%, 2% and 10% PLA to the soil respectively. To explain how non-biodegradable MPs such as LDPE contribute to the increase in soil DOC some researchers suggest that the reduction in bulk density, along with the increase in porosity and air circulation due to the presence of MPs particularly non-biodegradable ones leads to enhanced microbial activity. This, in turn, further degrades organic matter in the soil, resulting in an increase in DOC [4,80,81].

The contrasting behavior of MPs and PGPR observed between acidic and alkaline soils can be primarily attributed to variations in chemical properties of soils, microbial dynamics, and HM speciation. In acidic soils, lower pH enhances the solubility and bioavailability of cationic metals such as Cd^2+^ and Pb^2+^, whereas in alkaline soils, the formation of carbonate or hydroxide precipitates restricts their mobility [82]. The biodegradable PLA increased DOC and moderated soil pH, thereby promoting metal complexation and decreasing the activity of free metal ions [31,83,84]. In contrast, the nonpolar LDPE tended to lower pH and increase EC, which enhanced the bioavailability and plant uptake of HMs [70,85–87] Furthermore, inherent differences in microbial community structure and enzymatic activity between soil types may regulate these processes, influencing the combined behavior of MPs, PGPR, and HMs. Collectively, these mechanisms explain the soil pH–dependent responses observed in this study.

### 4.2. The effect of co-contamination of MPs and HMs on the maize growth

Overall, the outcomes of this study indicate that the concurrent presence of HMs contamination and MPs, particularly LDPE, exerts more detrimental effects on maize growth in both soil types. Several studies have also found that MPs exacerbate the negative impacts of HMs on plant growth [25,35,41,88].

In alkaline soil, the combined contamination of MPs and HMs reduced the dry weight of both maize shoots and roots compared to the control, with only LDPE treatments causing a significant decrease in plant biomass. In acidic soil, PLA, unlike LDPE, increased the plant’s dry weight. Wang et al. [83] reported similar findings, where 1% biodegradable MPs such as PLA and PBAT(Poly butylene adipate-co-terephthalate) boosted cabbage root dry weight by over 120%, without significantly affecting shoot weight. In contrast, Li et al. [31] found that PLA reduced plant dry weight in Cd-contaminated soil, while PE had no notable effect. These differences may be due to variations in soil conditions and the amounts of MPs used, which could have impacted soil properties, especially pH.

In both soil types, LDPE reduced leaf chlorophyll index, stem diameter, MSI, and RWC, with the lowest values observed in the 5% LDPE treatment. The effect of PLA on these indices was not significant in alkaline soil, though a decreasing trend was seen compared to the control. However, in acidic soil, PLA increased leaf chlorophyll index and stem diameter. This suggests that PLA was less toxic to plants than LDPE in both soil types. Furthermore, in acidic soil, PLA slightly enhanced certain growth parameters, such as stem diameter and chlorophyll index. Zeb et al. [35] found a decrease in chlorophyll content in lettuce leaves under combined polyester and Cd contamination, while Jia et al. [88] observed reduced chlorophyll levels in rapeseed exposed to high concentrations of Pb, Cu, and PE.

In both soil types, the use of PGPR increased the dry weight of shoots and roots. However, bacteria’s ability to remediate combined contamination of HMs and MPs was less effective compared to HMs contamination alone. In acidic soil, the combined contamination of HMs and PLA did not negatively affect PGPR performance, unlike the combination of HMs and LDPE. Similar findings were found by [40,41] who also reported PGPR’s role in reducing combined HMs and MPs contamination. Zhang et al. [42] noted that the combined contamination of Cd and PVC decreased the availability of essential soil nutrients like potassium, phosphorus, and ammonium nitrate, which reduced plant growth. However, PGPR application improved plant growth by enhancing soil mineral nutrient content. In this study, PGPR also improved several plant growth parameters, especially in alkaline soil. Importantly, no notable variation was found between the effectiveness of the two types of bacteria. Similar positive effects of PGPR on plant growth in soils contaminated with HMs have been reported by other researchers [36,74,75,89].

It should be noted that the performance of inoculated PGPR is inevitably influenced by the native soil microbial community. The introduced bacteria may engage in synergistic or antagonistic interactions with indigenous soil microorganisms, which can alter their overall impact on soil properties and plant growth [90,91]. The fact that PGPR improved certain plant growth and soil parameters in both soil types examined in this study indicates a degree of functional robustness. However, their effectiveness may vary across soils with different microbial backgrounds, as competition, cooperation, and niche overlap with native microorganisms can affect the establishment and expression of PGPR functions [92].

### 4.3. The effect of combined contamination of MPs and HMs on metal concentrations and transfer factor (TF)

In general, it can be concluded that the presence of LDPE, particularly at higher concentrations (5%), increased the concentration of HMs in plants in both soil types. The lowest values for shoot and root dry weight, chlorophyll index, stem diameter, MSI, and RWC in this treatment highlight the role of LDPE in elevating HMs concentration and its negative impact on maize growth. Another key finding is that PLA treatment reduced metal concentrations in plants in both soil types, suggesting that PLA may help alleviate the negative impacts of HMs contamination on plant growth.

In both alkaline and acidic soils, soil pH exhibited the strongest significant negative correlation with metal concentrations in both shoots and roots of maize (Fig 6), this indicates that soil pH is the main factor affecting metal concentrations in plants. A decrease in soil pH reduces the surface adsorption of HMs, increasing their bioavailability and, consequently, their uptake by plants [93,94]. Therefore, the observed increase in metal concentrations in maize tissue in the presence of LDPE can be attributed to its role in lowering soil pH which is likely attributable to changes in the activity of nitrifiers and the increased release of H^+^ [70]. On the other hand, several studies have noted that higher salinity levels reduce the capability of microplastics to attract contaminants, as ions in the solution occupy the MPs’ active adsorption sites [95,96]. Therefore, another potential reason for the increased bioavailability of HMs in the presence of LDPE may be the rise in EC caused by this polymer, which likely results from enhanced soil aeration and accelerated mineralization of organic matter in LDPE-amended soils [85]. (Fig 2C). Ondrasek et al. [86] and Wang et al. [87] observed that higher EC enhanced the availability of anions such as chloride (Cl^-^) and sulfate (${\text{SO}}_{\text{4}}^{\text{2}-}$), forming Cd-chloride and Cd-sulfate complexes, which increased the bioavailability of Cd. Ultimately, the relatively smooth and chemically inert surface of LDPE, characterized by a limited number of functional groups, reduces its capacity to adsorb metal ions compared to PLA. As a result, the concentration of free metals in the soil solution increases. The combination of these mechanisms provides a clear explanation for the enhanced metal uptake observed in plants exposed to LDPE treatment.

Ding et al. [67] and Huang et al. [97] reported increases in Cd bioavailability, while Chen et al. [98] and Hurley and Nizzetto, [99] observed an increase in Pb availability in the presence of PE contamination. Bethanis and Golia, [69] found that 5% PE addition increased Zn and Cd concentrations in lettuce roots by 26.6% and 11.2%, respectively, and in leaves by 21.1% and 10%. Similarly, Li et al. [11] reported that 10% PE reduced the surface adsorption of Ni and Cu in soil by 13.7% and 15.9%, respectively, leading to increased bioavailability and toxicity of these metals.

The reduction in metal concentrations in maize in treatments with PLA in both soil types may also be influenced by the elevation of soil pH due to PLA contamination. Partial degradation of PLA in soil, whether through hydrolysis or microbial activity, leads to the release of lactic acid monomers and oligomers [100]. These compounds are relatively water-soluble and serve as rapidly decomposable carbon sources for soil microorganisms, increasing soil DOC and potentially triggering a positive priming effect, which can alter the decomposition rate or pattern of native soil organic matter as well as the structure and functioning of the microbial community [101]. PLA can also increase the population of copiotrophic bacteria and alter the fungi-to-bacteria ratio [102]. These microbial changes directly affect enzymatic activities and nitrogen cycling pathways, and can modify the balance of H⁺ and OH⁻ ions, ultimately leading to changes in soil pH. Depending on conditions such as PLA particle size and concentration, soil type, buffering capacity, moisture content, and duration of exposure, these mechanisms may either increase or decrease soil pH [70,103]. In our study, PLA exhibited an increasing effect on soil pH, and this higher pH reduced the solubility of HMs. Wang et al. [83] reported a decline in Cd concentrations in cabbage shoots and roots in treatments with biodegradable PLA and PBAT, compared to PE and polypropylene (PP), attributing this reduction to the rise in soil pH resulting from these MPs. Another possible explanation for the reduced metal concentrations in plants treated with PLA is its rougher surface, higher quantity of oxygen-containing functional groups and greater surface activity and adsorption energy compared to LDPE (Fig. 1; S3 Table). These features likely enhance the adsorption of HMs on the surface of PLA, reducing their bioavailability. Liu et al. [95] noted that plastic particles with rougher surfaces can adsorb more pollutants. Chen et al. [28] found that an increase in oxygen-containing functional groups enhances the pollutant-adsorption potential of MPs. Lin et al. [104] reported that PLA has a higher capacity to adsorb HMs than PE, attributing this to its higher abundance of oxygen-containing functional groups, larger specific surface area, rougher surface, and lower crystallinity. Additionally, the increase in soil DOC in the presence of PLA, particularly in acidic soils, may reduce HMs availability, thereby lowering their concentration in plants. Zhao et al. [84] observed a significant negative correlation between soil DOC content and available Cd concentration, attributing this effect to the increased presence of C-O, C-C, and C = O functional groups following the rise in DOC levels. These functional groups are associated with HMs surface adsorption. Similarly, Yu et al. [105] indicated that DOC, a complex polymer containing aromatic and carboxyl groups, can adsorb HMs, thereby reducing their availability in the soil solution. Together, these processes explain why PLA treatments reduced HM availability and uptake in maize.

In both soil types and across all treatments, the transfer factor (TF) of metals was less than one, indicating that metals were more concentrated in the roots than in the aerial parts, suggesting metal stabilization in the roots. The presence of MPs further reduced this factor compared to the control, consistent with the findings of Li et al. [31], who observed a decrease in the TF value of Cd in the presence of high concentrations of PE and PLA. In contrast, Zhao et al. [30] reported no significant effect of PS and PP on the TF value of Cd.

## 5. Limitations of this study

This study was conducted under controlled pot conditions over a relatively short period, which may not fully reflect the complexity of long-term field environments. Nevertheless, this setup allowed precise control of experimental variables and detailed examination of factor interactions. Only a single microplastic particle size range (0.5–1 mm) and relatively high pollutant concentrations were tested; therefore, future studies should investigate a wider range of particle sizes and contaminant levels to achieve a more comprehensive understanding of ecological implications. Fresh and pristine microplastics were used to minimize compositional uncertainties, whereas weathered particles in natural environments may exhibit different behaviors. Moreover, the establishment of inoculated bacterial strains was not directly verified, and biochemical and molecular analyses, such as antioxidant enzyme activities and lipid peroxidation, were not performed due to laboratory and resource limitations. Future research incorporating direct bacterial quantification and detailed physiological assessments could provide deeper insights into plant responses under combined MPs–HMs stress.

## 6. Conclusion

This study examined the effects of two types of MPs, biodegradable PLA and non-biodegradable LDPE, on soil properties, maize growth, and chemical composition of it in alkaline and acidic soils contaminated with HMs. The findings underscore the importance of MPs type, concentration, and soil characteristics in determining the environmental impact of these pollutants. Polylactic acid, as a biodegradable polymer, increased soil pH, while LDPE lowered pH and raised EC. The impact of PLA on increasing soil DOC was more pronounced, particularly in acidic soil, compared to LDPE. The interaction between HMs and MPs varied significantly: LDPE enhanced the bioavailability and uptake of HMs in maize, while PLA reduced metal concentrations in plant tissues. The reduction in HMs toxicity in the presence of PLA can be ascribed to its effects on increasing soil pH and DOC, as well as its unique characteristics, including functional groups composition and surface morphology. Plant growth indices, including biomass, chlorophyll index, and MSI, were significantly suppressed by LDPE, particularly at higher concentrations, while PLA showed neutral to positive effects, particularly in acidic soils. Furthermore, the application of PGPR, including *P. fluorescens* and *A. lipoferum*, effectively reduced the harmful effects of MPs and HMs contamination. PGPR treatments notably improved biomass production, chlorophyll index, and soil DOC levels, demonstrating their potential to support sustainable agricultural practices under combined stress conditions. However, the interactions between inoculated PGPR and the indigenous soil microbial community warrant further investigation, as these complex biotic interactions could significantly modulate the observed outcomes in different agricultural soils. Present study highlights the potential of biodegradable MPs, like PLA to reduce the harmful effects of MPs contamination. However, because bioplastics are more easily broken down by UV radiation and soil microorganisms, they may generate additional MPs, which could have a more pronounced effect on soil characteristics, along with the structure and diversity of soil bacteria. Therefore, future research is highly recommended to assess the production of secondary MPs from PLA degradation and investigate other aspects of their impacts, particularly over longer time periods.

## Supporting information

S1 Table Two-way ANOVA significance levels for microplastics, bacteria, and their interactions in alkaline soil. (DOCX)

S2 Table Two-way ANOVA significance levels for microplastics, bacteria, and their interactions in Acidic soil. (DOCX)

S3 Table BET–BJH surface characterization parameters of PLA and LDPE microplastics. (DOCX)

S1 Data Data supporting this study. (XLSX)
