# Supplementary material for: Combined effects of heavy metals and microplastics on maize grown in acid and alkaline soils inoculated with plant growth promoting rhizobacteria
Source: PLoS One. 2025 Dec 30;20(12):e0338112. doi: 10.1371/journal.pone.0338112 (PMC12752957; doi:10.1371/journal.pone.0338112)
Supplement: S1 Table — (DOCX) [file pone.0338112.s001.docx]

S1 Table. Significance levels of MPs (type and concentration) (A), bacteria (B) and their interactions (A×B) on measured variables on a two-way ANOVA analysis for Alkaline soil, including F value and Partial Eta Squared ($\eta_{P}^{2}$).

|  | Source |  | A | |  |  |  | B | |  |  |  | A×B | |  |
| --- | --- | --- | --- | --- | --- | --- | --- | --- | --- | --- | --- | --- | --- | --- | --- |
|  |  | *df* | *F Value* | *P*  *Value* | $\eta_{P}^{2}$ |  | *df* | *F*  *Value* | *P*  *Value* | $\eta_{P}^{2}$ |  | *df* | *F Value* | *P Value* | $\eta_{P}^{2}$ |
| Shoot | dry weight | 4 | 27.85 | <.0001 | 0.79 |  | 2 | 58.47 | <.0001 | 0.80 |  | 8 | 2.28 | 0.049 | 0.38 |
| Root | dry weight | 4 | 40.31 | <.0001 | 0.84 |  | 2 | 93.16 | <.0001 | 0.86 |  | 8 | 2.43 | 0.037 | 0.39 |
|  | Chl | 4 | 3.74 | 0.014 | 0.33 |  | 2 | 176.64 | <.0001 | 0.92 |  | 8 | 0.81 | 0.60 | 0.18 |
|  | MSI | 4 | 34.69 | <.0001 | 0.82 |  | 2 | 6.59 | 0.0042 | 0.31 |  | 8 | 0.82 | 0.59 | 0.18 |
|  | RWC | 4 | 15.63 | <.0001 | 0.68 |  | 2 | 3.72 | 0.036 | 0.20 |  | 8 | 0.37 | 0.93 | 0.09 |
|  | SD | 4 | 5.13 | 0.0029 | 0.41 |  | 2 | 7.92 | 0.0017 | 0.35 |  | 8 | 0.54 | 0.82 | 0.13 |
| Soil | DOC | 4 | 8.97 | <.0001 | 0.54 |  | 2 | 74.81 | <.0001 | 0.83 |  | 8 | 2.84 | 0.018 | 0.43 |
|  | EC | 4 | 3.31 | 0.023 | 0.31 |  | 2 | 10.18 | 0.0004 | 0.40 |  | 8 | 2.19 | 0.058 | 0.37 |
|  | pH | 4 | 19.51 | <.0001 | 0.72 |  | 2 | 14.5 | <.0001 | 0.49 |  | 8 | 0.19 | 0.99 | 0.05 |
| TF | Pb | 4 | 3.51 | 0.018 | 0.32 |  | 2 | 6.15 | 0.0058 | 0.29 |  | 8 | 1.17 | 0.35 | 0.24 |
|  | Cd | 4 | 10.12 | <.0001 | 0.57 |  | 2 | 7.62 | 0.0021 | 0.34 |  | 8 | 1.44 | 0.22 | 0.28 |
|  | Zn | 4 | 0.60 | 0.66 | 0.07 |  | 2 | 5.93 | 0.0067 | 0.28 |  | 8 | 0.20 | 0.99 | 0.05 |
|  | Ni | 4 | 2.62 | 0.055 | 0.26 |  | 2 | 5.96 | 0.0066 | 0.28 |  | 8 | 0.37 | 0.93 | 0.09 |
| Pb | Shoot | 4 | 39.00 | <.0001 | 0.84 |  | 2 | 14.26 | <.0001 | 0.49 |  | 8 | 1.12 | 0.38 | 0.23 |
|  | Root | 4 | 30.79 | <.0001 | 0.80 |  | 2 | 0.66 | 0.53 | 0.04 |  | 8 | 0.23 | 0.98 | 0.06 |
| Cd | Shoot | 4 | 58.26 | <.0001 | 0.89 |  | 2 | 2.33 | 0.115 | 0.13 |  | 8 | 1.07 | 0.41 | 0.22 |
|  | Root | 4 | 12.06 | <.0001 | 0.62 |  | 2 | 20.53 | <.0001 | 0.58 |  | 8 | 1.07 | 0.41 | 0.22 |
| Zn | Shoot | 4 | 5.37 | 0.0022 | 0.42 |  | 2 | 9.68 | 0.0006 | 0.39 |  | 8 | 0.38 | 0.92 | 0.09 |
|  | Root | 4 | 14.49 | <.0001 | 0.66 |  | 2 | 0.07 | 0.93 | 0.00 |  | 8 | 0.16 | 0.99 | 0.04 |
| Ni | Shoot | 4 | 26.54 | <.0001 | 0.78 |  | 2 | 0.40 | 0.67 | 0.03 |  | 8 | 0.36 | 0.94 | 0.09 |
|  | Root | 4 | 36.47 | <.0001 | 0.83 |  | 2 | 14.36 | <.0001 | 0.49 |  | 8 | 0.79 | 0.62 | 0.17 |
| Abbreviations: A: MPs (type and concentration), B: bacteria, MSI: membrane stability index, RWC: relative water content, DOC: dissolved organic carbon, EC: electrical conductivity; TF; transfer factor; Chl: chlorophyll (SPAD reading); SD: stem diameter, $\eta_{P}^{2}$: partial Eta Squared (Interpretation, X< 0.01: negligible, 0.01 ≤ X < 0.06: small effect, 0.06 ≤ X < 0.14: Medium effect, X ≥ 0.14: large effect). | | | | | | | | | | | | | | | |
