# Supplementary material for: Combined effects of heavy metals and microplastics on maize grown in acid and alkaline soils inoculated with plant growth promoting rhizobacteria
Source: PLoS One. 2025 Dec 30;20(12):e0338112. doi: 10.1371/journal.pone.0338112 (PMC12752957; doi:10.1371/journal.pone.0338112)
Supplement: S2 Table — (DOCX) [file pone.0338112.s002.docx]

S2 Table. Significance levels of MPs (type and concentration) (A), bacteria (B) and their interactions (A×B) on measured variables on a two-way ANOVA analysis for Acidic soil, including F value and Partial Eta Squared (partial η²).

|  | Source |  | A | |  |  |  | B | |  |  |  | A×B | |  |
| --- | --- | --- | --- | --- | --- | --- | --- | --- | --- | --- | --- | --- | --- | --- | --- |
|  |  | *df* | *F Value* | *P*  *Value* | $\eta_{P}^{2}$ |  | *df* | *F*  *Value* | *P*  *Value* | $\eta_{P}^{2}$ |  | *df* | *F Value* | *P Value* | $\eta_{P}^{2}$ |
| Shoot | dry weight | 4 | 59.57 | <.0001 | 0.89 |  | 2 | 52.21 | <.0001 | 0.78 |  | 8 | 2.54 | 0.031 | 0.40 |
| Root | dry weight | 4 | 26.60 | <.0001 | 0.78 |  | 2 | 27.51 | <.0001 | 0.65 |  | 8 | 2.60 | 0.028 | 0.41 |
|  | Chl | 4 | 7.18 | 0.0004 | 0.49 |  | 2 | 5.12 | 0.012 | 0.25 |  | 8 | 0.07 | 0.99 | 0.02 |
|  | MSI | 4 | 25.17 | <.0001 | 0.77 |  | 2 | 0.44 | 0.65 | 0.03 |  | 8 | 0.11 | 0.99 | 0.03 |
|  | RWC | 4 | 12.32 | <.0001 | 0.62 |  | 2 | 0.70 | 0.50 | 0.04 |  | 8 | 0.24 | 0.98 | 0.06 |
|  | SD | 4 | 14.51 | <.0001 | 0.66 |  | 2 | 2.72 | 0.082 | 0.15 |  | 8 | 0.15 | 0.99 | 0.04 |
| Soil | DOC | 4 | 20.89 | <.0001 | 0.74 |  | 2 | 88.46 | <.0001 | 0.86 |  | 8 | 2.33 | 0.045 | 0.38 |
|  | EC | 4 | 3.76 | 0.014 | 0.33 |  | 2 | 4.12 | 0.026 | 0.22 |  | 8 | 0.82 | 0.59 | 0.18 |
|  | pH | 4 | 98.18 | <.0001 | 0.93 |  | 2 | 4.27 | 0.023 | 0.22 |  | 8 | 0.12 | 0.99 | 0.03 |
| TF | Pb | 4 | 3.75 | 0.014 | 0.33 |  | 2 | 1.62 | 0.21 | 0.10 |  | 8 | 0.69 | 0.70 | 0.15 |
|  | Cd | 4 | 2.64 | 0.053 | 0.26 |  | 2 | 0.08 | 0.92 | 0.01 |  | 8 | 0.71 | 0.68 | 0.16 |
|  | Zn | 4 | 4.36 | 0.0067 | 0.37 |  | 2 | 0.81 | 0.46 | 0.05 |  | 8 | 0.44 | 0.89 | 0.11 |
|  | Ni | 4 | 1.21 | 0.33 | 0.14 |  | 2 | 4.66 | 0.017 | 0.24 |  | 8 | 1.24 | 0.31 | 0.25 |
| Pb | Shoot | 4 | 32.20 | <.0001 | 0.81 |  | 2 | 8.31 | 0.013 | 0.36 |  | 8 | 2.75 | 0.021 | 0.42 |
|  | Root | 4 | 39.08 | <.0001 | 0.84 |  | 2 | 0.10 | 0.91 | 0.01 |  | 8 | 0.11 | 0.99 | 0.03 |
| Cd | Shoot | 4 | 51.12 | <.0001 | 0.87 |  | 2 | 6.78 | 0.0037 | 0.31 |  | 8 | 0.44 | 0.89 | 0.11 |
|  | Root | 4 | 38.41 | <.0001 | 0.84 |  | 2 | 4.97 | 0.014 | 0.25 |  | 8 | 1.46 | 0.21 | 0.28 |
| Zn | Shoot | 4 | 28.97 | <.0001 | 0.79 |  | 2 | 2.51 | 0.098 | 0.14 |  | 8 | 0.43 | 0.89 | 0.10 |
|  | Root | 4 | 25.67 | <.0001 | 0.77 |  | 2 | 6.75 | 0.0038 | 0.31 |  | 8 | 0.73 | 0.66 | 0.16 |
| Ni | Shoot | 4 | 14.31 | <.0001 | 0.66 |  | 2 | 2.41 | 0.11 | 0.14 |  | 8 | 0.47 | 0.87 | 0.11 |
|  | Root | 4 | 17.38 | <.0001 | 0.70 |  | 2 | 12.77 | <.0001 | 0.46 |  | 8 | 1.06 | 0.42 | 0.22 |
| Abbreviations: A: MPs (type and concentration), B: bacteria, MSI: membrane stability index, RWC: relative water content, DOC: dissolved organic carbon, EC: electrical conductivity; TF; transfer factor; Chl: chlorophyll (SPAD reading); SD: stem diameter, $\eta_{P}^{2}$: partial Eta Squared (Interpretation, X< 0.01: negligible, 0.01 ≤ X < 0.06: small effect, 0.06 ≤ X < 0.14: Medium effect, X ≥ 0.14: large effect). | | | | | | | | | | | | | | | |
