# Supplementary material for: Combined effects of heavy metals and microplastics on maize grown in acid and alkaline soils inoculated with plant growth promoting rhizobacteria
Source: PLoS One. 2025 Dec 30;20(12):e0338112. doi: 10.1371/journal.pone.0338112 (PMC12752957; doi:10.1371/journal.pone.0338112)
Supplement: S3 Table — (DOCX) [file pone.0338112.s003.docx]

S3 Table. Summary of BET–BJH surface characterization parameters for PLA and LDPE microplastics

| Parameter | Unit | PLA | LDPE |
| --- | --- | --- | --- |
| BET surface area (as, BET) | m²·g⁻¹ | 1.32 | 1.01 |
| BET constant (C) | — | 10.31 | 6.29 |
| Total pore volume (p/p₀=0.99) | cm³·g⁻¹ | 0.0836 | 0.0427 |
| Mean pore diameter | nm | 103.16 | 168.61 |
| BJH peak pore radius (rp,peak) | nm | 7.99 | 4.69 |
| BJH surface area (ap) | m²·g⁻¹ | 6.66 | 3.52 |
